# Comparison of PCR, Nested PCR, and RT-LAMP for Rapid Detection of Feline Calicivirus Infection in Clinical Samples

**DOI:** 10.3390/ani14162432

**Published:** 2024-08-22

**Authors:** Piyamat Khamsingnok, Witsanu Rapichai, Amonpun Rattanasrisomporn, Oumaporn Rungsuriyawiboon, Kiattawee Choowongkomon, Jatuporn Rattanasrisomporn

**Affiliations:** 1 Graduate Program in Animal Health and Biomedical Sciences, Faculty of Veterinary Medicine, Kasetsart University, Bangkok 10900, Thailand; piyamat.kha@ku.th; 2 Department of Companion Animal Clinical Sciences, Faculty of Veterinary Medicine, Kasetsart University, Bangkok 10900, Thailand; tswitsanu@gmail.com; 3 Department of Biochemistry, Faculty of Science, Kasetsart University, Bangkok 10900, Thailand; fsciktc@ku.ac.th; 4 Interdisciplinary of Genetic Engineering and Bioinformatics, Graduate School, Kasetsart University, Bangkok 10900, Thailand; fgraapr@ku.ac.th; 5 Department of Veterinary Technology, Faculty of Veterinary Technology, Kasetsart University, Bangkok 10900, Thailand; cvtopr@ku.ac.th

**Keywords:** PCR, Nested PCR, RT-LAMP, feline calicivirus, cat

## Abstract

**Simple Summary:**

Feline calicivirus (FCV) is a major cause of upper respiratory tract (URT) disease in cats that can lead to acute or subacute illness. It is highly contagious and widespread in the feline population, with many cats being asymptomatic carriers. However, FCV infection can also cause various clinical problems, resulting in high morbidity rates. Unfortunately, existing diagnostic techniques are not always sufficiently quick or specific enough to detect the virus, making it difficult to diagnose and treat. The current study compared the diagnostic abilities of different methods—polymerase chain reaction (PCR), nested PCR, and reverse transcription loop-mediated isothermal amplification (RT-LAMP)—to detect FCV in clinical samples from cats. The development of rapid and sensitive diagnostic methods is crucial for timely healing, reducing the risk of severe symptoms, and preventing the spread of viruses.

**Abstract:**

Feline calicivirus (FCV) is a highly contagious virus that causes upper respiratory tract disease, commonly known as cat flu. It is widely distributed worldwide and poses a major threat to feline health. Therefore, it is essential to find an efficient and rapid method for detecting FCV. In this study, the colorimetric reverse transcription loop-mediated isothermal amplification (RT-LAMP) assay, using neutral red as an indicator, was developed and validated to target the ORF2 gene of FCV for the first time. Additionally, the study compared the diagnostic abilities of polymerase chain reaction (PCR), nested PCR, and RT-LAMP assays for detecting FCV in clinical samples. The optimized RT-LAMP amplification was carried out at 56.3 °C. The technique visually detected FCV within 70 min, with a limit of detection of 14.3 × 10^1^ copies/µL, and showed no cross-reactivity with other feline pathogens. Out of 54 oropharyngeal swab samples, 17 tested positive for FCV using both nested PCR and RT-LAMP, while only one tested positive using conventional PCR. The positivity rate was higher with nested PCR and RT-LAMP (31.48%) compared to conventional PCR (1.85%). Consequently, these results demonstrated the effectiveness of the colorimetric RT-LAMP assay developed in this study as an alternative for diagnosing FCV in cats.

## 1. Introduction

Feline calicivirus (FCV, genus *Vesivirus*, family *Caliciviridae*) is a common viral pathogen that causes upper respiratory tract (URT) disease or cat flu [1,2]. FCV has a single-stranded positive-sense RNA genome, which encodes three open reading frames (ORFs): ORF1, ORF2, and ORF3 [3,4,5]. ORF1 encodes a large polyprotein that includes the viral polymerase, ORF2 encodes the major structural capsid protein VP1, and ORF3 encodes the minor structural protein VP2. Based on amino acid sequence alignment and antigenic analysis, ORF2 can be divided into six distinct regions (A–F) [6,7], containing both conserved and variable sequences. Region A is associated with the leader capsid (LC) proteins. Regions B, D, and F are relatively conserved, while regions C and E are highly divergent among FCV isolates [8]. Region E is particularly immunodominant and comprises three parts: the 5′ hypervariable region (5′ HVR-E), the conserved central region (Cons-E), and the 3′ hypervariable region (3′ HVR-E). This variable region contains the major B-cell epitopes, making it a target for virus-neutralizing antibodies [7,9]. The length of FCV is approximately 7.7 kb [8,10,11]. Cats can be infected with FCV through various routes such as the nasally, orally, or conjunctively [12,13]. Additionally, the virus is often carried to susceptible cats by human handlers; however, humans are not susceptible to infection [12]. The incubation period is 2–6 days [14]. Many infected cats may not show any noticeable symptoms. Additionally, some infected felines still carry the virus or have a mild and undetectable form of the disease [15]. The clinical features present in FCV-infected cats are fever, rhinitis, conjunctivitis, stomatitis, glossitis, ulcers in the mouth, and discharge from the nose and eyes [16,17]. Cats may also experience limping syndrome as a result of FCV infection [15,18]. In severe cases, cats could experience pulmonary edema and interstitial pneumonia [19,20]. The mortality rate for very young kittens can be as high as 30% [14]. In addition, FCV is found in approximately 50% of cats showing signs of acute upper respiratory disease. A recent study reported that FCV infections were observed in Thai cats, with an overall prevalence of 46.7% [21]. Many cats that recover from the virus remain persistently infected and can shed it from their oropharynx for several years, and in some cases, this could continue for their entire life [22].

The gold standard method for diagnosing FCV infections is virus isolation. However, this method is not suitable for routine diagnosis due to its requirements for high-skill techniques, increased labor, and time [10,23,24]. In recent years, molecular techniques have been developed, such as conventional PCR, nested PCR, real-time RT-PCR (RT-qPCR), and nanoparticle-assisted PCR (NanoPCR), providing faster and more reliable tools for virus detection. Although several PCR methods have been reported for the detection of FCV [21,25,26,27,28], the high genetic variability of the virus poses challenges in maintaining diagnostic sensitivity.

Nested PCR is a variation of standard PCR in which two pairs of PCR primers are used (instead of one pair) to target a single locus [29]. The first set of primers amplifies the target fragment in a conventional PCR reaction. The second set of primers, known as “nested primers”, binds inside the first PCR product to enable the amplification of a second PCR product, which is shorter than the first. The nested PCR method serves to increase assay sensitivity by re-amplifying the target from a template previously enriched by the first PCR. However, non-target sequences that were amplified non-specifically in the first PCR are not re-amplified in the second reaction. As a result, nested PCR substantially improves both specificity and detection sensitivity compared to conventional PCR [30,31]. However, these PCR methods have limitations and require sophisticated equipment and skills. Additionally, the PCR products must be detected using gel electrophoresis, which increases the time and cost of detection [32].

Loop-mediated isothermal amplification (LAMP) was introduced by Notomi and colleagues as a method for DNA amplification [33]. LAMP reactions can be carried out in isothermal conditions without advanced laboratory equipment, such as a dry block heater or a water bath, which means there is no time loss caused by temperature changes. The LAMP amplification relies on a set of 4–6 primers, specially designed to recognize 6–8 distinct regions of a target gene, resulting in high efficiency and specificity [33]. The primer set consists of two outer primers (F3 and B3), two inner primers (forward inner primer (FIP) and backward inner primer (BIP)), and loop primers (loop forward and loop backward). The FIP primer consists of the F1 complementary sequence (F1c) and the F2 sense sequence, while the BIP primer consists of the B1 complementary sequence (B1c) and the B2 sense sequence. This technique can detect both DNA and RNA targets as it is able to detect RNA targets through the reverse transcription-LAMP (RT-LAMP) reaction. By using *Bst* DNA/RNA polymerase, it is possible to simultaneously carry out reverse transcription and DNA amplification at a constant temperature, completing the entire process in just 60 min [34]. The LAMP products can be visually detected by intercalating fluorescent dye [35], the turbidity of white Mg-pyrophosphate precipitate [36,37], agarose gel electrophoresis, colorimetric methods visible to the naked-eye, and real-time fluorimeters. Hence, this technique offers major advantages, including high specificity, ease of operation, rapid reaction time, excellent sensitivity, and affordability. Furthermore, this technique has been widely applied to identify various pathogens in feline clinical specimens such as feline coronavirus (FCoV) [38], feline immunodeficiency virus (FIV) [39], *Tritrichomonas foetus* [40], and *Toxoplasma gondii* [41]. However, no known reports have been published on the detection of FCV in cats using an RT-LAMP assay.

The objective of the current study was to create a new method for detecting FCV in cats, using a RT-LAMP-neutral red assay. The RT-LAMP method was tested on clinical samples and the results were compared to the conventional diagnostic methods (conventional PCR and nested PCR assays). This was the first time that the colorimetric RT-LAMP assay has been used for detecting FCV.

## 2. Materials and Methods

### 2.1. Ethics Statement

The study obtained ethical approval from the Institutional Animal Care and Use Committee of Kasetsart University, Bangkok, Thailand (Protocol code ACKU66-VET-087) and was conducted according to the guidelines of the Declaration of Helsinki. Additionally, sample collection was authorized by the cat’s owner. 

### 2.2. Construction of Positive Control Plasmids

In order to construct recombinant plasmid (pTA-FCV-ORF2), a purified DNA fragment of FCV ORF2 gene (678 bp), using primers CaliAF and CaliAR reported by Yi and colleagues [42], was cloned into the T&A^TM^ cloning vector according to the manufacturer’s instruction (Yeastern Biotech, New Taipei City, Taiwan) and transformed into competent cells, *Escherichia coli* DH5α (Yeastern Biotech, New Taipei City, Taiwan). Transformants carrying the recombinant plasmid were grown on LB agar (Himedia, Mumbai, India) containing 100 µg/mL ampicillin (T.P. Drug Laboratories (1969), Bangkok, Thailand), 20 mg/mL X-gal (Thermo Fisher Scientific, Saint Louis, MO, USA), and 1M IPTG (Vivantis, Selangor Darul Ehsan, Malaysia) at 37 °C for 16–18 h. Only white colonies were selected to test colony PCR with M13 primers according to the manufacturer’s instructions (Yeastern Biotech, New Taipei City, Taiwan). Positive colony having inserted gene was inoculated in LB medium containing 100 µg/mL ampicillin and incubated at 37 °C and 200 rpm for 16–18 h. Then, the recombinant plasmid was then purified using an E.Z.N.A. Plasmid DNA Mini Kit I (Omega Bio-Tek, Norcross, GA, USA). To serve as the positive control for the RT-LAMP assay, the DNA concentration and quality were assessed using a NanoDrop spectrophotometer (Thermo Scientific, Wilmington, NC, USA), and verified using DNA sequencing (Macrogen, Seoul, Republic of Korea).

### 2.3. Conventional PCR and Nested PCR

Initially, FCV cDNA synthesis was performed using 2 µL of viral RNA extracted from clinical samples using the E.Z.N.A. Viral RNA Kit (Omega Bio-Tek, Norcross, GA, USA) as templates in a RevertAid RT Reverse Transcription Kit (Thermo Scientific, Wilmington, NC, USA). The resulting FCV cDNA was further utilized as a template for conventional PCR amplification. In the first stage of conventional PCR, the FCV ORF2 gene was amplified using the FCV-Cali1 and FCV-Cali2 primers listed in Table 1, which produced a DNA band of approximately 927 bp. The amplification reaction was conducted in a total volume of 25 µL, containing 12.5 µL of 2X AccuStart^TM^ II GelTrack^TM^ PCR SuperMix (Quantabio, Beverly, MA, USA), 0.6 µM of each primer, 1 µL cDNA, and 8.5 µL RNase-free water. Amplification was carried out using a Thermocycler (Bio-Rad, Hercules, CA, USA) involving an initial denaturation at 95 °C for 5 min, followed by 35 cycles of denaturation at 95 °C for 45 s, annealing at 57 °C for 45 s, and extension at 72 °C for 30 s, with a final extension at 72 °C for 10 min. The PCR amplicons were templated for a second reaction using the FCV-Cali3 and FCV-Cali4 nested primers (Table 1), which yielded a DNA band of approximately 477 bp. The second stage of nested PCR was performed by amplifying 1 µL of the first-stage PCR products in a total volume of 25 µL of PCR reaction mix containing all the same ingredients as the previous PCR solution, with the exception of using the nested primers (FCV-Cali3 and FCV-Cali4) instead of the first set. The reaction mixture was amplified for 35 cycles under the same conditions as the conventional PCR. The PCR products were stained using loading dye (Applied Biological Materials Inc., Vancouver, BC, Canada) and then loaded onto a 1.5% (*w*/*v*) agarose gel for electrophoresis. The visualization was carried out using the BluPAD LED Transilluminator (BIO-HELIX, New Taipei City, Taiwan).

### 2.4. RT-LAMP Primer Design

**Figure 1:**
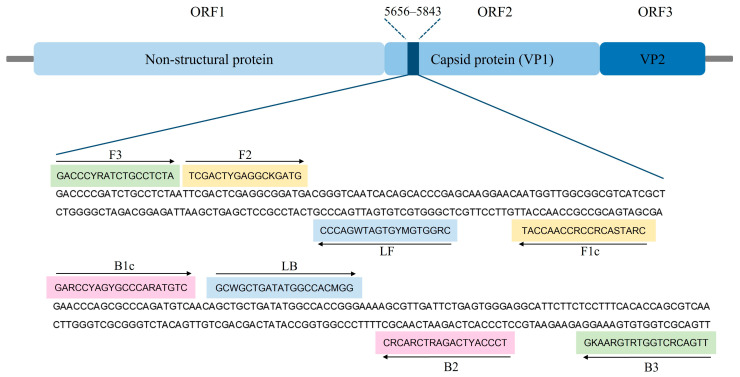
Genome map locations of FCV (GenBank accession No. M86379) and binding sites of RT-LAMP primers in ORF2 gene sequence. The sequences marked in green, yellow, pink, and blue represent two outer primers (F3 and B3), two inner primers (F1c-F2 (FIP) and B1c-B2 (BIP)), and two loop primers (LF and LB), respectively. Arrows indicate the extension direction.

Due to the presence of the most-highly conserved sequence of FCV [43], the RT-LAMP primers set was designed to target the ORF2 gene (FCV-vaccine strains with GenBank database with accession numbers AF479590, KM111170, and M86379) using the PrimerExplorer V5 software (http://primerexplorer.jp/lampv5e/index.html (accessed on 9 May 2024)). To ensure the specificity of the RT-LAMP primers, in silico analysis was conducted with sequences of feline infectious peritonitis virus (FIP) (AY994055), feline immunodeficiency virus (FIV) (M25381), feline panleukopenia virus (FPV) (MG924893), feline leukemia virus (FeLV) (MT129531), and feline herpesvirus (FHV) (NC_013590) using BLAST search. The primers were synthesized by Macrogen (Macrogen, Seoul, Republic of Korea), and the sequences of the primer set are provided in Table 1, while the genomic structure of FCV and the position of the RT-LAMP primers are shown in Figure 1.

### 2.5. RT-LAMP Assay

The RT-LAMP reaction was performed in a total volume of 25 μL, consisting of 2.5 µL 10X ammonium sulfate buffer (100 mM (NH_4_)_2_SO_4_, 100 mM KCl, and 10% Tween 20 pH 8.8), 7 µL 25 mM MgCl_2_, 4 µL 10 mM dNTPs solution mixture (Biotechrabbit, Berlin, Germany), 2.5 µL 10X RT-LAMP primer mix (1.6 µM each of FIP and BIP, 0.2 µM each of F3 and B3 primers, 0.4 µM each of Loop F and Loop B primers), 1 µL 40 U/µL RiboLock RNase inhibitor (Thermo Scientific, Wilmington, NC, USA), 1 µL 2.5 mM Neutral Red (NR) (Invitrogen, Waltham, MA, USA), 0.4 µL 8 U/µL *Bst* DNA/RNA polymerase (New England Biolabs, Ipswich, MA, USA), 5.6 µL RNase-free water (Apsalagen, Bangkok, Thailand), and 1 µL RNA template with pH adjusted to ~8.8 with 1N NaOH. The RT-LAMP reaction was incubated in a thermocycler (Bio-Rad, Hercules, CA, USA) at 56.3 °C for 70 min and then the reaction was terminated by holding at 85 °C for 5 min. After the isothermal reaction, the RT-LAMP products were detected based on naked-eye observation of color changes, where the positive reaction turned pink, while the negative reaction remained yellow. 

The optimum conditions for the RT-LAMP assay were determined by optimizing the key parameters of amplification temperatures (50–60 °C), amplification times (10, 20, 30, 40, 50, 60, 70, 80, or 90 min), MgCl_2_ concentrations (4, 6, 7, or 8 mM), and dNTPs concentrations (1.4, 1.6, 1.8, or 2.0 mM). Furthermore, to confirm the visual RT-LAMP assay, the fluorescence values were compared and analyzed throughout the entire course of the reaction based on real-time RT-LAMP fluorescent assay.

### 2.6. Validation of RT-LAMP Assay

The specificity of the RT-LAMP assay with a primer set was verified using an individual DNA or RNA sample extracted from five different feline viral species: feline coronavirus (FCoV), FeLV, FHV, FIV, and FPV. RNA extracted from uninfected CRFK cells (CRFK—ATCC^®^ Number: CCL-94TM, Manassas, VA, USA) and epithelial cells from the oral swab of a healthy cat (representative feline cells), as well as whole blood (representative feline blood cells), were also tested as internal controls. 

The sensitivity of the RT-LAMP assay was compared with conventional PCR and nested PCR assays based on the limit of detection (LOD) values using 10-fold serial dilutions of the pTA-FCV-ORF2. Both conventional PCR and nested PCR were examined using 10-fold serial dilutions of the synthetic FCV-ORF2 gene, which was assembled from synthetic oligonucleotides (Thermo Fisher Scientific, Saint Louis, MO, USA). The copy number of the DNA concentration was calculated using the following equation [44,45]: Number of copies (copies µL^−1^) = (concentration of nucleotide (ng µL^−1^) × 6.022 × 10^23^)/(length of nucleotide (bp) × 1 × 10^9^ × 650). RNase-free water was used as a no-template control (NTC), and all assays were performed in triplicate.

### 2.7. Application of RT-LAMP Assay in Clinical Samples

The performance of the RT-LAMP assay was evaluated using 54 clinical samples collected between August 2022 and September 2023 at the Kasetsart Veterinary Teaching Hospital, Bangkok, Thailand. Of these, 48 were obtained from oropharyngeal swabs of cats with upper respiratory tract disease showing signs of fever, rhinitis, conjunctivitis, stomatitis, glossitis, ulcers in the mouth, and discharge from the nose and eyes, while 6 were obtained from healthy cats. Oropharyngeal samples were collected in 250 µL of 1X phosphate-buffered saline with a pH of 7.4. After collection, the samples were immediately used for RNA extraction using the E.Z.N.A. Viral RNA Kit (Omega Bio-Tek, Norcross, GA, USA), and all samples were subsequently stored at −80 °C to maintain their integrity and stability until further analysis. The RT-LAMP amplification was performed under optimal conditions. To avoid non-specific amplification due to residual heat, the color changes were observed by the naked eye immediately after the heating process ended. A positive result was indicated by a change in the yellow reagent to pink through naked-eye observation. Additionally, all RT-LAMP products were confirmed based on electrophoresis on a 2% (*w*/*v*) agarose gel, followed by staining with loading dye. Positive reactions exhibited a DNA ladder-like pattern.

### 2.8. Sequencing and Analysis

Three positive samples were purified using Expin™ Combo (GeneAll Biotechnology, Seoul, Republic of Korea) and then sent for capillary electrophoresis sequencing (Macrogen, Seoul, Republic of Korea). The sequencing data were analyzed for similarity using the BLASTN software version 2.15.0 (http://www.ncbi.nlm.nih.gov (accessed on 9 May 2024)) in the NCBI database to confirm the results. Furthermore, the nucleotide sequences were compared for similarity using the BioEdit software version 7.2 (Informer Technologies Inc., Los Angeles, CA, USA). The nucleotide sequences of the samples were compared to the FCV reference sequence from NCBI (GenBank accession No. NC001481).

### 2.9. Statistical Analysis

A chi-squared test was applied to examine differences between the comparative variables. The *p*-value was used to determine whether there were any significant differences, with significance tested at the *p* < 0.05 level. The statistical analyses were conducted using the SPSS Statistics software version 26 (SPSS Inc., Chicago, IL, USA). The sensitivity, specificity, positive predictive value (PPV), and negative predictive value (NPV) of RT-LAMP were calculated using a 2 × 2 table, with each nested PCR assay as the comparator.

## 3. Results

### 3.1. Optimization of RT-LAMP Assay

The optimum temperature for the RT-LAMP assay was determined by incubating the RT-LAMP reaction at varied temperatures (50–60 °C) for 60 min. As shown in Figure S1, the pink color appearance in each tube of reactions showed the same color shade. From the real-time RT-LAMP analysis, the relative fluorescent signal curve of the positive reaction rapidly appeared at 56.3 °C. Thus, 56.3 °C was chosen as the optimum temperature. At the optimum temperature, the effect of amplification times on RT-LAMP reactions was determined by testing times in the range of 10–90 min. Figure S2 shows that the amplification pink color appeared as quickly as 40 min, with naked eye inspection identifying the most-intense pink color appearing at 70 min. Thus, 70 min was selected for further work using the RT-LAMP assay. The effect of the MgCl_2_ concentration on RT-LAMP assay was determined by testing different concentrations (4, 6, 7, and 8 mM). Figure S3 illustrates that a distinct pink coloration was observable at 7 mM MgCl_2_, which corresponded to the initial peak in the fluorescent signals. Hence, 7 mM MgCl_2_ was selected as the optimal concentration for the RT-LAMP assay. The effect of dNTPs concentration was determined by varying concentrations (1.4, 1.6, 1.8, and 2.0 mM). Figure S4 shows that dNTPs at the concentration of 1.6 mM had the most-distinctive pink color. The result of the 1.6 mM dNTPs tube was also confirmed by the corresponding fluorescent curve, which validated 1.6 mM as the optimal concentration. In summary, the key parameters for the optimal conditions of the RT-LAMP assay for FCV detection were amplification at 56.3 °C for 70 min, 7 mM MgCl_2_, and 1.6 mM dNTPs.

### 3.2. Specificity Evaluation of RT-LAMP Assay

**Figure 2:**
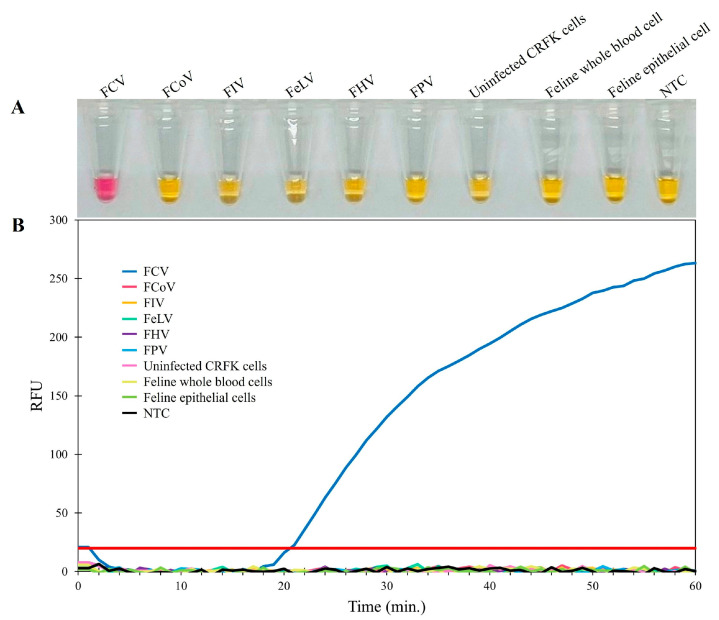
Specificity determination of the RT-LAMP assay. (**A**) Colorimetric RT-LAMP results are indicated by neutral red and visualized by the eye, where positive reactions are pink in color, while negative reactions are yellow. (**B**) Evaluation using real-time fluorescent RT-LAMP assay.

The specificity of the RT-LAMP assay was determined using the DNA or RNA samples extracted from five feline viruses (FCV, FCoV, FeLV, FHV, FIV, and FPV). The internal controls consisted of DNA extracted from uninfected CRFK cells and epithelial cells from the oropharyngeal swab of a healthy cat (representative feline cells), as well as whole blood (representative feline blood cells), all of which were also tested. The results revealed that the color change from yellow to pink in the visual inspection could only be observed for FCV, and no RT-LAMP products were detected in the reactions from other feline viruses, internal controls, or negative controls (Figure 2A). Thus, real-time fluorescent RT-LAMP assay (Figure 2B) was only positive for FCV (represented by the blue amplification line). The lack of any cross-reactions with these feline viruses indicated that the primers used in the RT-LAMP assay for FCV detection had specificity and could be used for the detection of clinical samples.

### 3.3. Assessment of Sensitivity of Conventional PCR, Nested PCR, and RT-LAMP Assays

**Figure 3:**
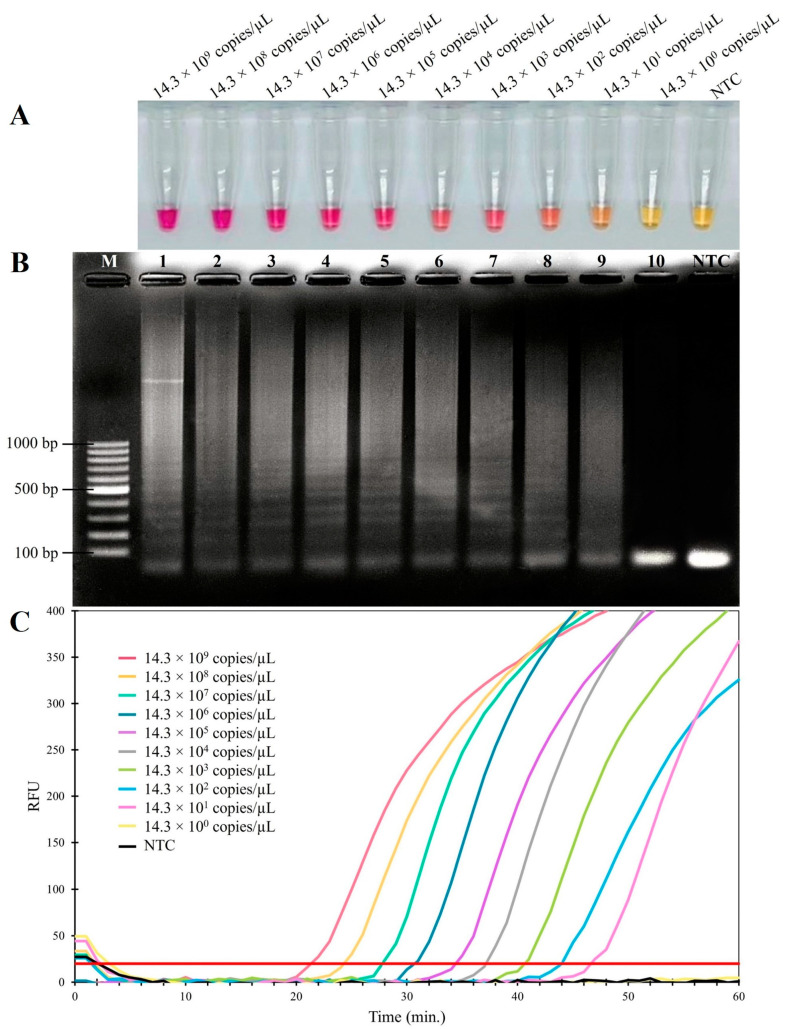
Sensitivity of RT-LAMP assay for detecting FCV. (**A**) Colorimetric RT-LAMP results are indicated by neutral red and visualized by the eye, where positive reactions are pink in color, while negative reactions are yellow. (**B**) Results from agarose gel electrophoresis showing the amplification products from the RT-LAMP assay. Lane M; 100 bp ladder DNA Marker (Vivantis, Selangor Darul Ehsan, Malaysia) (**C**) Evaluation using real-time fluorescent RT-LAMP assay.

**Figure 4:**
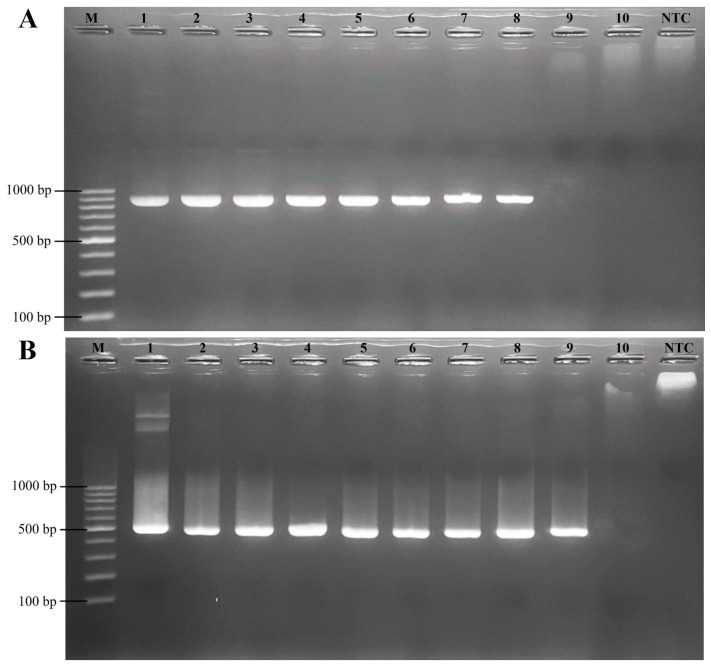
Sensitivity of conventional PCR and nested PCR assays for detecting FCV. (**A**) Evaluation using conventional PCR. (**B**) Evaluation using nested PCR. Lane M; 100 bp ladder DNA Marker (Vivantis, Selangor Darul Ehsan, Malaysia), lanes 1–10; 11.8 × 10^9^ copies/μL, 11.8 × 10^8^ copies/μL, 11.8 × 10^7^ copies/μL, 11.8 × 10^6^ copies/μL, 11.8 × 10^5^ copies/μL, 11.8 × 10^4^ copies/μL, 11.8 × 10^3^ copies/μL, 11.8 × 10^2^ copies/μL, 11.8 × 10^1^ copies/μL, and 11.8 × 10^0^ copies/µL (10-fold serial dilutions), respectively. Lane NTC; no template control as a negative control.

The sensitivity of the RT-LAMP assay for FCV was established on a 10-fold serial dilution of the pTA-FCV-ORF2 in the RT-LAMP assay, ranging from 14.3 × 10^9^ to 14.3 × 10^0^ copies/μL. As shown in Figure 3, the results indicated that the detection limit was 14.3 × 10^1^ copies/μL for both the visual inspection and the real-time RT-LAMP fluorescent assay. In addition, the sensitivity levels of the conventional PCR and nested PCR assays were assessed using 10-fold serial dilutions of the FCV-ORF2 synthetic gene, ranging from 11.8 × 10^9^ to 11.8 × 10^0^ copies/μL. The conventional PCR assay produced a detection limit of 11.8 × 10^2^ copies/μL (Figure 4A), while the nested PCR assay produced a detection limit of 11.8 × 10^1^ copies/μL (Figure 4B). As summarized in Table 2, these findings thus indicated that the RT-LAMP and nested PCR assays were 10-fold more sensitive than conventional PCR assays for the detection of FCV.

### 3.4. Evaluation of RT-LAMP Assay for Detection of FCV in Clinical Samples

The efficacy of the RT-LAMP assay was compared with that of conventional PCR and nested PCR assays by examining 54 clinical samples from the oropharyngeal swabs of cats (Table S1). Of these, 17 samples exhibited positive results of DNA amplification, showing a pink color after the completion of the RT-LAMP reaction, resulting in a positive rate of 31.48% (17/54), as shown in Table 3. These results were consistent with those from the nested PCR. With conventional PCR, only one sample was positive (1.85%). There was no significant difference in the positive rates between RT-LAMP and nested PCR (*p* > 0.05), but it was significantly higher than that of conventional PCR (*p* < 0.05). The clinical sensitivity, specificity, PPV, and NPV of RT-LAMP were all 100% when nested PCR was used as the reference method in this study.

**Figure 5:**
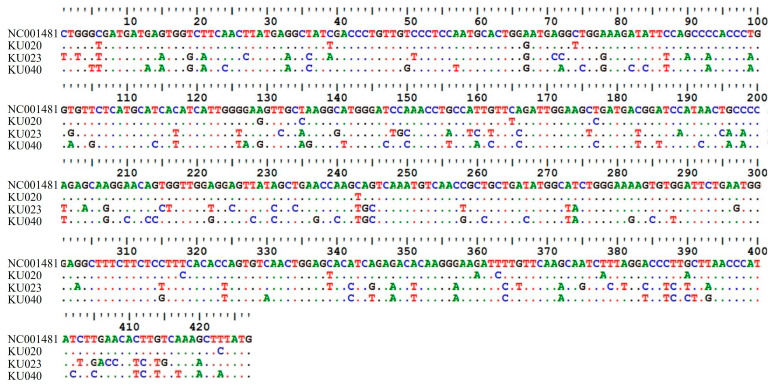
Comparison of nucleotide sequences between the FCV reference sequence from NCBI (GenBank accession. No. NC001481) and three FCV isolate samples, with sequence similarity in range 79.6–96.2%.

To confirm true positives, three positive samples (KU020, KU023, and KU040) were subjected to sequence analysis. The sequencing results for these three samples revealed a high degree of nucleotide identity to FCV sequences available in the GenBank database. Sample KU020 had 98.15% sequence similarity to the feline calicivirus isolate FCV_CU32 (MH561191), sample KU023 had 83.78% similarity to the FCV strain F65 (AF109465), and sample KU040 demonstrated 85.50% similarity to the FCV isolate HRB23 (MW804431). Additionally, the FCV reference sequence from NCBI (GenBank accession No. NC001481) was compared with the three samples for the 427 bp DNA fragments. The results of the sequence alignment were in the range of 79.6–96.2% (Figure 5). Furthermore, the sequence data were submitted to NCBI GenBank with accession numbers PP265922, PP265923, and PP265924.

## 4. Discussion

FCV is a highly contagious pathogen widely distributed globally and is a major threat to the health of felines [12,21,23]. Although the virus has been identified and separated in felines in multiple countries [25,27,46,47], there are still insufficient data regarding its prevalence in cat populations, specifically in Thailand. Phongroop et al. combined reverse-transcription quantitative PCR and high-resolution melting (RT-qPCR-HRM) assay to diagnose the genotype of FCV in cats naturally infected with the virus [48]. However, the RNA genome of FCV is highly variable, which could result in RT-PCR methods producing false negatives [10,27]. In addition, notably, the RT-qPCR-HRM assay has certain limitations, including the length, GC content, and integrity of the double-stranded DNA. Performing HRM analysis requires specific dyes, and real-time PCR instruments equipped with suitable software. Additionally, personal skill and expertise are necessary to perform the procedure correctly [49].

The current study was the first to present a colorimetric RT-LAMP-neutral red assay designed to target the ORF2 gene for detecting FCV in cats. Other studies have identified the ORF2 gene as a conserved region suitable for diagnostic purposes [21,31,42]. As a result, selecting the most conserved region for the primer design was a powerful feature of the development of the current method that has the potential to detect FCV. Displacement activity of the *Bst* DNA polymerase strand eliminates the need for DNA denaturation in LAMP assays that can be carried out isothermally without advanced laboratory equipment [33,50]. The use of isothermal polymerases with reverse transcription activity allowed for the amplification of RNA targets. LAMP products are amplified DNA molecules that can be easily detected by using specialized equipment, such as a real-time fluorimeter or real-time turbidimeter, or using naked-eye observation of color changes. The key factor for visualizing the LAMP results in the current study was the neutral red pH-sensitive dye. Another study used a pH-sensitive indicator in a lightly buffered LAMP solution to induce a color shift due to the hydrogen ions produced during DNA biosynthesis [51]. A major change in pH values from initially alkaline to acidic can detect LAMP amplification using pH-sensitive indicator dyes. Neutral red is a chromogenic agent that can be added to LAMP reaction mixtures before DNA amplification without inhibition [52]. As shown in the current study, it enables clear visualization of major color changes associated with positive and negative results using the naked eye in daylight.

The current study reported detection limits of RT-LAMP (14.3 × 10^1^ copies/μL) and nested PCR (11.8 × 10^1^ copies/μL) that were significantly lower than those of conventional PCR (11.8 × 10^2^ copies/μL), suggesting that both RT-LAMP and nested PCR had superior sensitivity in detecting low concentrations of the target nucleic acids compared to conventional PCR. The increased sensitivity of RT-LAMP can be attributed to its unique mechanism that involves a set of specially designed primers. These primers target specific regions in the DNA sequence, resulting in more efficient and precise amplification. Similarly, nested PCR, with its two-step amplification process, increases the likelihood of detecting low-abundance targets by minimizing non-specific amplification [29]. Additionally, the nested PCR primers used in the current study were referenced from Marsilio et al., due to their reported sensitivity (13 × 10^1^ copies/μL) being higher than that of other diagnostic techniques such as virus isolation in cell culture and RT-PCR [28]. Our RT-LAMP assay has demonstrated sensitivity levels comparable to those reported in previous studies. The clinical sample analysis showed that the positive rates of FCV detected using RT-LAMP (31.48%) and nested PCR (31.48%) were higher than that using conventional PCR (1.85%). These results could be attributed to the differences in analytical sensitivity (LODs) among the three assays. Samples with low viral loads may yield negative results with conventional PCR but positive results with the RT-LAMP and nested PCR assays. Thus, the analysis of clinical samples revealed consistent results between RT-LAMP and nested PCR. Notably, all the positive samples were collected from oropharyngeal swabs of cats with URT disease symptoms such as fever, rhinitis, conjunctivitis, stomatitis, glossitis, ulcers in the mouth, and discharge from the nose and eyes. Apart from FCV, this syndrome has been associated with at least four other pathogens—feline herpesvirus type 1 (FHV-1), *Mycoplasma felis*, *Chlamydophila felis*, and *Bordetella bronchiseptica* [1,15,53,54,55,56,57,58,59,60]. This finding underscores the complexity of diagnosing URT diseases in cats, as multiple pathogens can contribute to similar clinical manifestations. The consistent results between RT-LAMP and nested PCR suggested that both methods are effective for detecting FCV, a common pathogen in such cases. However, future studies should explore the inclusivity and sensitivity of these molecular assays in detecting a broader spectrum of pathogens associated with feline upper respiratory disease. Such insights would further validate their utility in clinical practice for accurate and timely diagnosis, facilitating appropriate treatment and management strategies for affected cats. However, the nested PCR assay takes around 7 h from RNA extraction to detection, whereas the RT-LAMP assay developed in the current study completed the same process in about 2 h. Furthermore, the results were easily visible to the naked eye when using a neutral red pH-sensitive indicator dye. In contrast, a conventional and nested PCR often requires more than 30 min to determine results, using gel electrophoresis that involves a time-consuming process that includes preparing the gel (agarose or polyacrylamide) and conducting the electrophoresis.

A limitation of the current study was that there is no gold standard for comparing molecular methods in diagnosing FCV. Virus isolation may fail due to low virus numbers in the sample, virus inactivation during transport, or the presence of antibodies in extracellular fluids that prevent virus replication in vitro [23,61,62]. Virus isolation is not commonly used in clinical diagnosis owing to its high cost and the lengthy time requirement of several days to 2 weeks [10,23,24]. Consequently, sequencing was adopted as a confirmatory test in the current study due to its reliability and ability to provide detailed genetic information. Therefore, three randomly selected positive samples (KU020, KU023, and KU040) were subjected to sequence analysis. That analysis revealed a high degree of nucleotide identity based on using BLAST in the NCBI database to confirm the FCV results, in the range of 83.78–98.15%. This analysis supported the accuracy of the molecular detection methods in the current study, showing consistent results that aligned with FCV genetic sequences available in public databases. Despite these challenges, molecular methods, such as RT-LAMP, offer rapid and sensitive alternatives for FCV detection, overcoming some of the limitations associated with virus isolation. Therefore, the RT-LAMP assay is more appropriate than PCR for rapid detection of FCV detection, especially for routine diagnosis, because of its high specificity, sensitivity, and visually detectable results. Although the LAMP assay has several benefits, it also has some limitations: (1) detecting LAMP requires utilizing 4–6 primers of varying lengths per target, complicating the design process and impacting detection accuracy; (2) LAMP necessitates extra primers and costly enzymes, rendering it pricier than conventional PCR though, nonetheless, it can be economically and straightforwardly conducted with just a heat block; (3) due to the LAMP products being multiple-loop DNA molecules with a cauliflower-like structure, the technique is unsuitable for cloning or sequencing [63,64,65]; and (4) LAMP is sensitive to cross-contamination, meaning that material in aerosols and contamination of samples with exogenous genetic material can affect the results. To avoid contamination, it is important to ventilate the room and analyze samples separately [66]. Thus, operators must be vigilant regarding sample contamination risks and adhere to specific sterility protocols.

Despite these considerations, the study confirmed RT-LAMP to be highly accurate, demonstrating 100% sensitivity, specificity, PPV, and NPV compared to nested PCR, which served as the reference method. This underscored the potential of RT-LAMP as an effective diagnostic tool for FCV, emphasizing its advantages in terms of speed and accuracy over PCR methods in clinical and veterinary settings. Future research should continue to validate and optimize RT-LAMP protocols while addressing its practical challenges to further enhance its utility and reliability in routine diagnostic applications.

## 5. Conclusions

A colorimetric RT-LAMP-neutral red assay targeting the ORF2 gene has been developed to detect FCV in cats for the first time. The entire process required only 70 min of operation in an isothermal environment (56.3 °C), and the amplification effect was visible to the naked eye. Notably, there was no need to remove the reaction tube lid for result observation, providing a certain degree of protection against aerosol contamination during RT-LAMP amplification. Accordingly, the colorimetric RT-LAMP-neutral red assay described in the current study could serve as a practical molecular tool to conduct diagnostic purposes for FCV under simple laboratory conditions.

## Figures and Tables

**Table 1:** Primers used in study for FCV amplification targeting ORF2 gene.

Primer Name	Sequence (5′-3′)	Ref.
FCV-F3 ^a^	GACCCYRATCTGCCTCTA	This work
FCV-B3 ^a^	TTGACRCTGGTRTGRAAKG	
FCV-FIP ^a^ (F1c-F2)	CRATSACRCCRCCAACCAT-TCGACTYGAGGCKGATG	
FCV-BIP ^a^ (B1c-B2)	GARCCYAGYGCCCARATGTC-TCCCAYTCAGARTCRACRC	
FCV-LF ^a^	CRGGTGMYGTGATWGACCC	
FCV-LB ^a^	GCWGCTGATATGGCCACMGG	
FCV-Cali1 ^b^	CAACCTGCGCTAACGTGCTTA	[24]
FCV-Cali2 ^b^	CAGTGACAATACACCCAGAAG	
FCV-Cali3 ^c^	TGGTGATGATGAATGGGCATC	
FCV-Cali4 ^c^	ACACCAGAGCCAGAGATAGA	

Degenerated bases were used according to IUPAC nomenclature: R (A/G), W (A/T), Y (C/T), M (A/C), K (G/T), and S (C/G). ^a^ RT-LAMP; ^b^ Conventional PCR; ^c^ Nested PCR.

**Table 2:** Detection Limits of RT-LAMP, nested PCR, and conventional PCR.

Method	Detection Limit
RT-LAMP	14.3 × 10^1^ copies/μL
Nested PCR	11.8 × 10^1^ copies/μL
Conventional PCR	11.8 × 10^2^ copies/μL

**Table 3:** Comparison of RT-LAMP, nested PCR, and conventional PCR assays for detecting FCV in 54 clinical samples.

Result	No. of Samples with Indicated Results Based on		
	RT-LAMP	Nested PCR	Conventional PCR
Positive	17	17	1
Negative	37	37	53
Total	54	54	54

RT-LAMP positive rate of FCV: 17/54 = 31.48%; Nested PCR positive rate of FCV: 17/54 = 31.48%; Conventional PCR positive rate of FCV: 1/54 = 1.85%.

## Data Availability

The data presented in this study are available within the article. Raw data supporting this study are available from the corresponding author.

## Supplementary Materials

The following supporting information can be downloaded at: https://www.mdpi.com/article/10.3390/ani14162432/s1, Figure S1. Optimization of amplification temperatures for RT-LAMP assay. (A) Colorimetric RT-LAMP results indicated by neutral red and visualized by eye, where positive reactions are pink in color, while negative reactions are yellow. NTC (no template control as a negative control). (B) Evaluation using real-time fluorescent RT-LAMP assay. Figure S2. Optimization of incubation time for RT-LAMP assay. NTC (no template control as a negative control). Figure S3. Optimization of MgCl2 concentrations for RT-LAMP assay. (A) Colorimetric RT-LAMP results indicated by neutral red and visualized by eye, where positive reactions are pink in color, while negative reactions are yellow. NTC (no template control as a negative control). (B) Evaluation using real-time fluorescent RT-LAMP assay. Figure S4. Optimization of dNTPs concentrations for RT-LAMP assay. (A) Colorimetric RT-LAMP results indicated by neutral red and visualized by eye, where positive reactions are pink in color, while negative reactions are yellow. NTC (no template control as a negative control). (B) Evaluation using real-time fluorescent RT-LAMP assay. Table S1. Clinical samples were tested with RT-LAMP, nested PCR, and conventional PCR assays.
