# Supplementary material for: Comparison of PCR, Nested PCR, and RT-LAMP for Rapid Detection of Feline Calicivirus Infection in Clinical Samples
Source: Animals (Basel). 2024 Aug 22;14(16):2432. doi: 10.3390/ani14162432 (PMC11350671; doi:10.3390/ani14162432)
Supplement: Supplementary file 1 [file animals-14-02432-s001.zip › Supplementary_RT-LAMP_FCV-1.pdf]

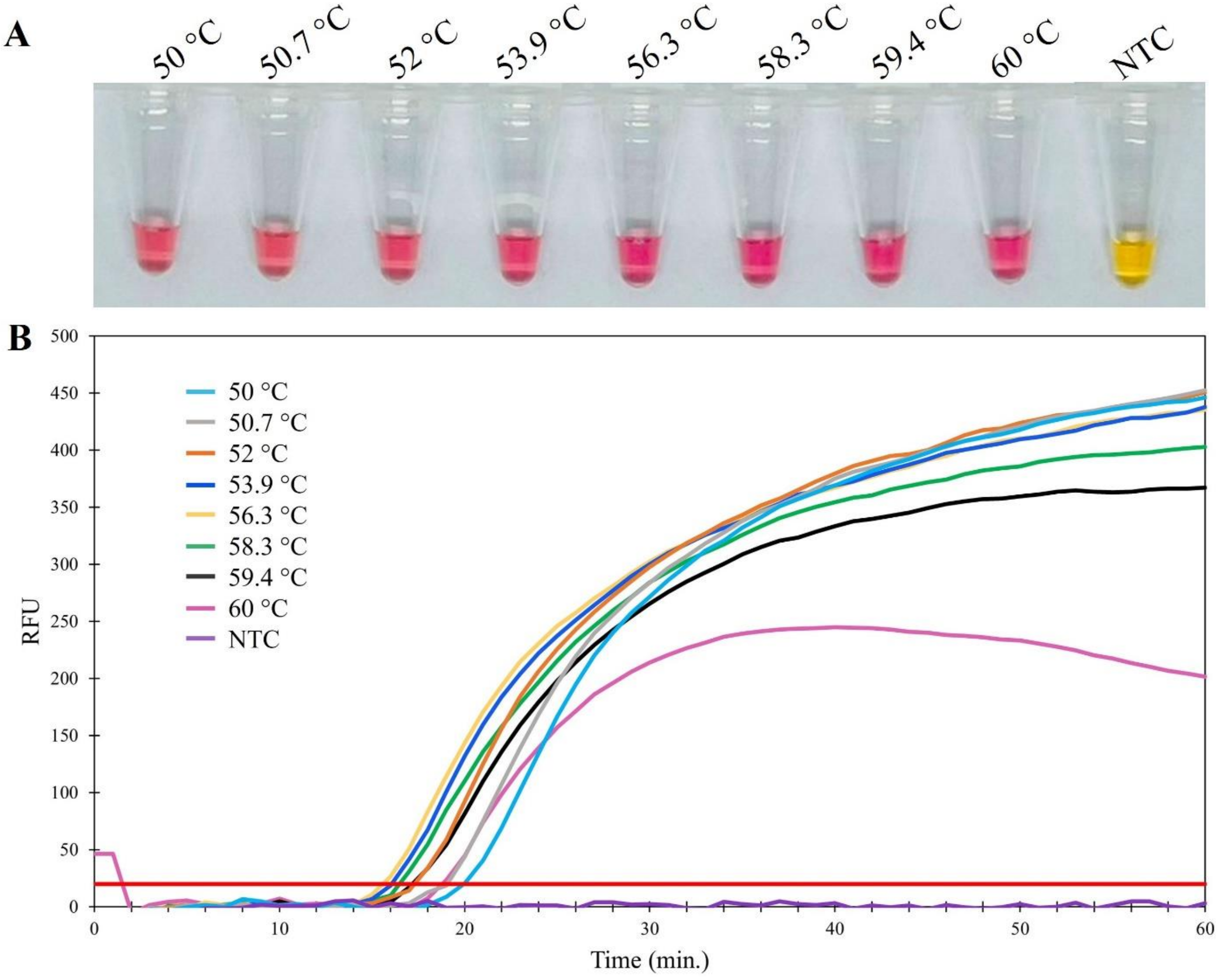

Figure S1. Optimization of amplification temperatures for RT-LAMP assay. (A) Colorimetric RT-LAMP results indicated by Neutral Red and visualized by eye, where positive reactions are pink in color, while negative reactions are yellow. NTC (no template control as a negative control). (B) Evaluation using real-time fluorescent RT-LAMP assay.

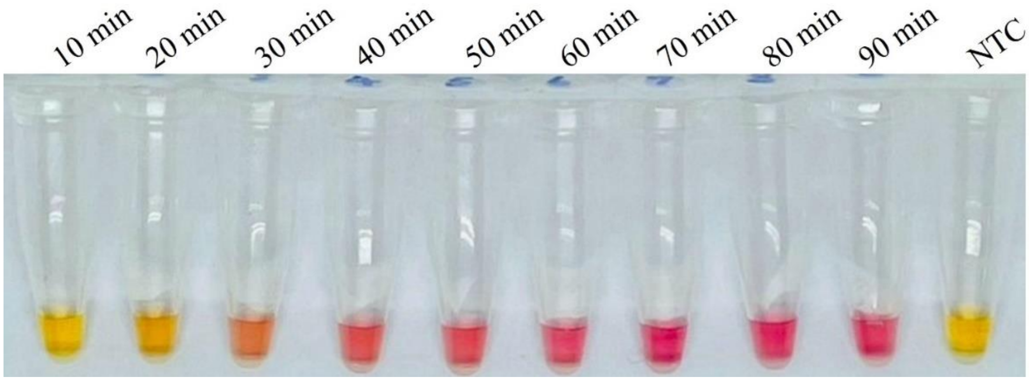

Figure S2. Optimization of incubation time for RT-LAMP assay. NTC (no template control as a negative control).

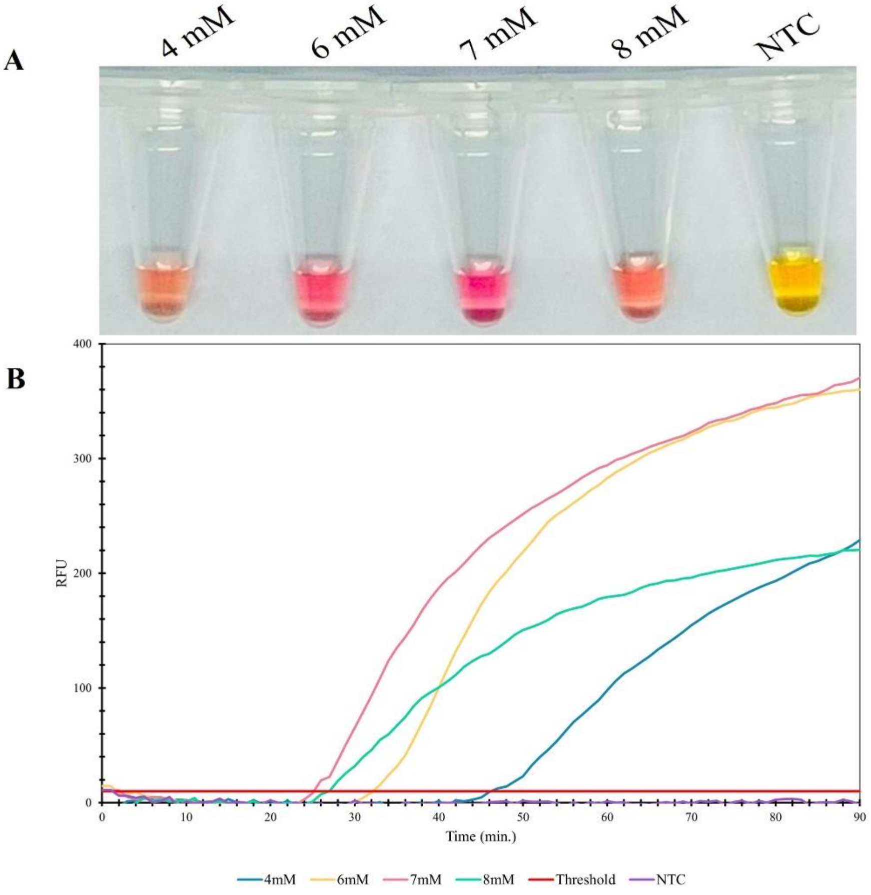

Figure S3. Optimization of $MgCl_2$ concentrations for RT-LAMP assay. (A) Colorimetric RT-LAMP results indicated by Neutral Red and visualized by eye, where positive reactions are pink in color, while negative reactions are yellow. NTC (no template control as a negative control). (B) Evaluation using real-time fluorescent RT-LAMP assay.

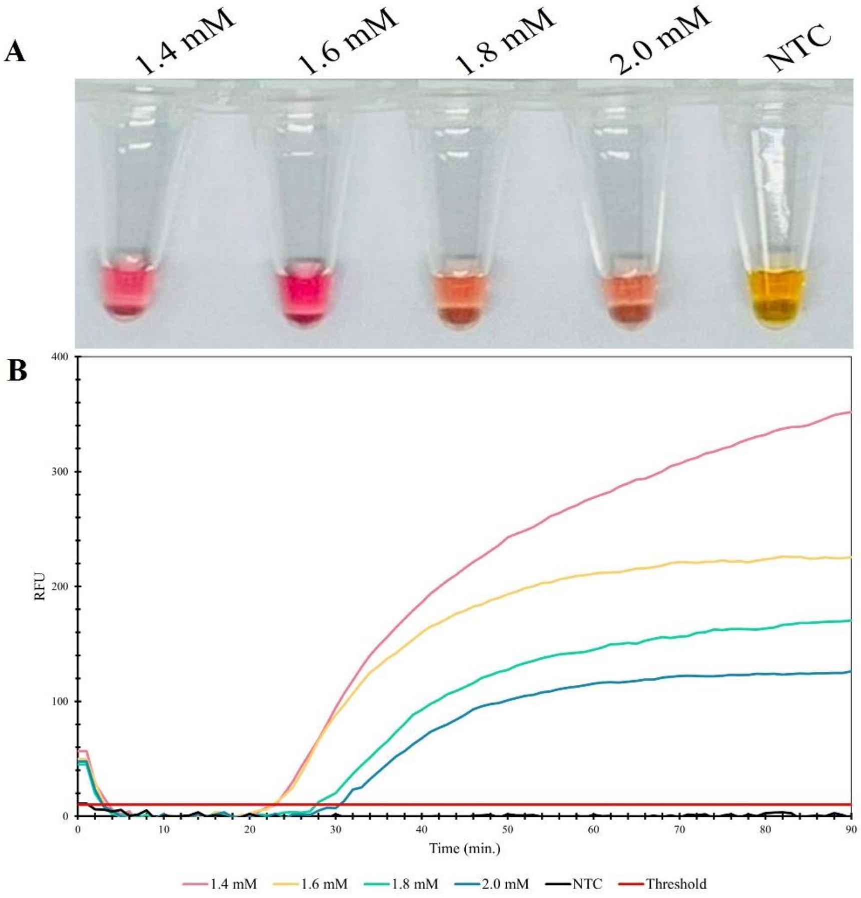

Figure S4. Optimization of dNTPs concentrations for RT-LAMP assay. (A) Colorimetric RT-LAMP results indicated by Neutral Red and visualized by eye, where positive reactions are pink in color, while negative reactions are yellow. NTC (no template control as a negative control). (B) Evaluation using real-time fluorescent RT-LAMP assay.

Table S1. Clinical samples were tested with RT-LAMP, nested PCR, and conventional PCR assays.

Name	Result		
	RT-LAMP	nested PCR	conventional PCR
KU001	+	+	-
KU002	+	+	-
KU003	+	+	-
KU004	+	+	-
KU005	-	-	-
KU006	+	+	-
KU007	-	-	-
KU008	-	-	-
KU009	-	-	-
KU010	-	-	-
KU011	-	-	-
KU012	-	-	-
KU013	-	-	-
KU014	-	-	-
KU015	-	-	-
KU016	-	-	-
KU017	-	-	-
KU018	+	+	-
KU019	+	+	-
KU020	+	+	-
KU021	+	+	-
KU022	-	-	-
KU023	+	+	+
KU024	-	-	-

Name	Result		
	RT-LAMP	nested PCR	conventional PCR
KU025	-	-	-
KU026	-	-	-
KU027	+	+	-
KU028	-	-	-
KU029	-	-	-
KU030	-	-	-
KU031	+	+	-
KU032	+	+	-
KU033	-	-	-
KU034	-	-	-
KU035	+	+	-
KU036	-	-	-
KU037	-	-	-
KU038	-	-	-
KU039	-	-	-
KU040	+	+	-
KU041	-	-	-
KU042	-	-	-
KU043	-	-	-
KU044	-	-	-
KU045	+	+	-
KU046	-	-	-
KU047	+	+	-
KU048	-	-	-

Name	Result		
	RT-LAMP	nested PCR	conventional PCR
KU049	-	-	-
KU050	-	-	-
KU051	-	-	-
KU052	-	-	-
KU053	-	-	-
KU054	-	-	-
